# Supplementary material for: The interactome of intact mitochondria by cross-linking mass spectrometry provides evidence for coexisting respiratory supercomplexes
Source: Mol Cell Proteomics. 2017 Dec 8;17(2):216–32. doi: 10.1074/mcp.RA117.000470 (PMC5795388; doi:10.1074/mcp.RA117.000470)
Supplement: Supplemental Data [file supp_RA117.000470_133922_0_supp_23978_fzffwf.zip › spectra_annotation/mito_DR_spectra_annotation/309-1-3-1-20-1.pdf]

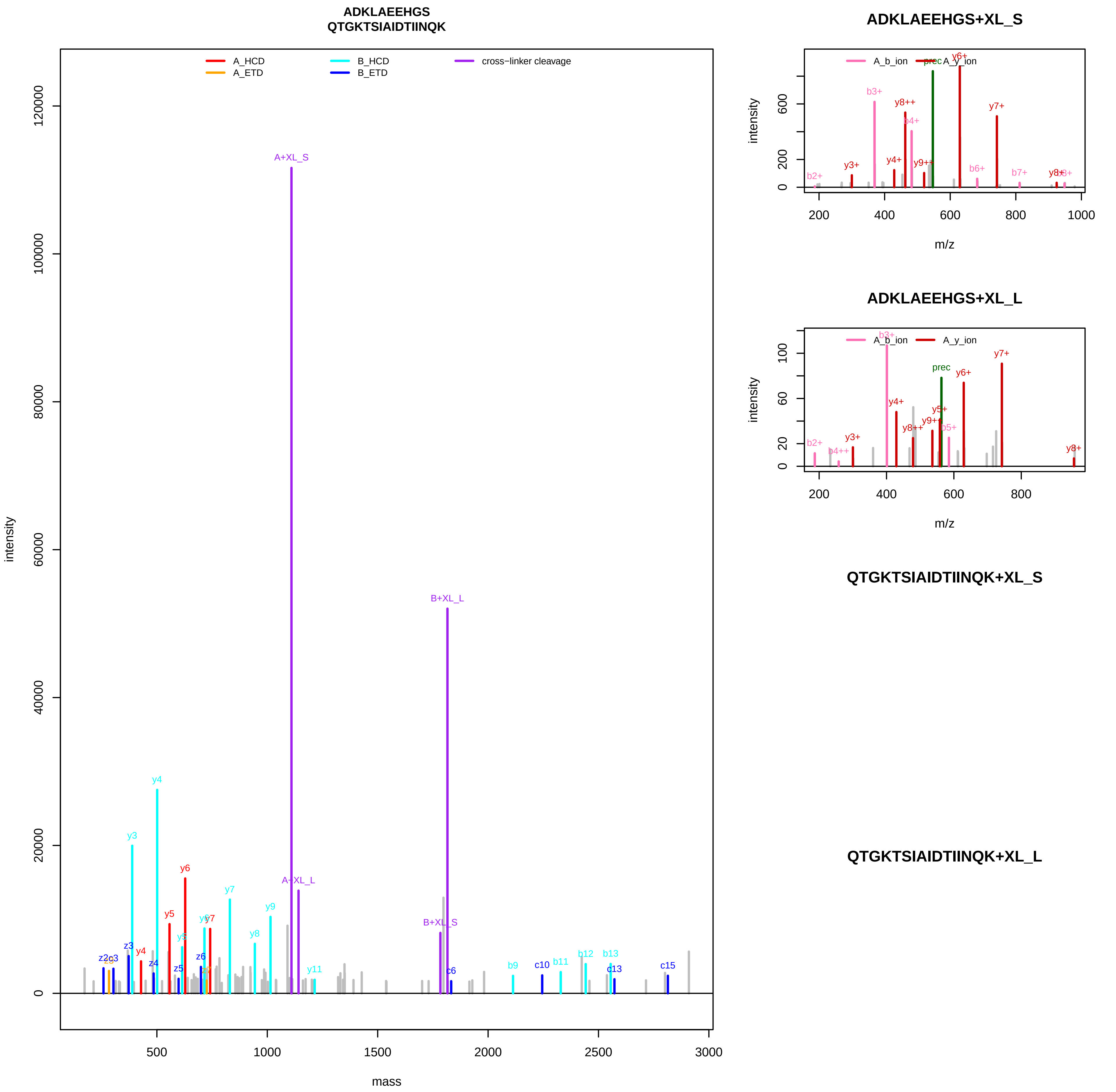
ADKLAEEHGS
QTGKTSIAIDTIINQK
A_HCD
A_ETD
B_HCD
B_ETD
cross−linker cleavage
A+XL_S
B+XL_L
A+XL_L
B+XL_S
intensity
mass
0
20000
40000
60000
80000
100000
120000
500
1000
1500
2000
2500
3000
y3
y4
y5
y6
y7
y8
y9
y11
z2
c3
z3
z4
z5
z6
c6
b9
c10
b11
b12
b13
c13
c15
ADKLAEEHGS+XL_S
A_b_ion
A_y_ion
prec
b2+
b3+
b4+
b6+
b7+
y3+
y4+
y6+
y7+
y8++
y9++
y8+
m/z
200
400
600
800
1000
ADKLAEEHGS+XL_L
b4++
b5+
y5+
QTGKTSIAIDTIINQK+XL_S
QTGKTSIAIDTIINQK+XL_L
